# The effect of patient-led goal setting approach combined with pain neuroscience education or manual therapy in patients with chronic low back pain: protocol for a parallel-group randomized controlled trial

**DOI:** 10.1186/s13063-023-07617-1

**Published:** 2023-09-08

**Authors:** Sahar Soheili, Amir Letafatkar, Mahdi Hosseinzadeh

**Affiliations:** 1 Department of Biomechanics and Sport Injuries, Faculty of Physical Education and Sport Sciences, Kharazmi University, Tehran, Iran; 2 Sports Injury and Corrective Exercises, Kharazmi University, Tehran, Iran; 3 Department of Sport Injuries and Corrective Exercises, Sport Sciences Research Institute, No. 3, 5Th Alley, Miremad Street, Motahhari Street, Tehran, 1587958711 Iran

**Keywords:** Patient-led goal setting, Pain neuroscience education, Chronic low back pain, Manual therapy

## Abstract

**Background:**

Low back pain (LBP) is a multifactorial disorder associated with a high range of physical and psychological burden on the society. Patient-led goal-setting approach has shown potential effects for improving chronic LBP. However, there are few studies investigating its benefits when combined with a supplementary intervention. This paper, therefore, presents a protocol for a randomized control trial (RCT) to study the effect of a patient-led goal-setting approach combined with pain neuroscience education (PNE) or manual therapy (MT) among patients with chronic LBP.

**Methods:**

A total of 105 patients suffering from chronic LBP will be recruited via flyers displayed in hospitals and universities, and those meeting the study’s criteria will randomly be allocated into a patient-led goal-setting approach with the PNE group, and/or with the MT program group, and/or a control group. The primary outcomes will be the pain intensity and disability. Secondary outcomes include quality of life, depression, anxiety and stress, fear avoidance beliefs, kinesophobia, pain self-efficacy, catastrophic pain, neurophysiology of pain, and central sensitivity. All the outcomes will be recorded at 2 months after receiving the treatment as post-test sessions and after 4 and 12 months as follow-up sessions. The Ethics Committee in Research at Sport Sciences Research Institute of Iran approved the protocol of this trial (IR.SSRC.REC.1400.084). Written, informed consent to participate will be obtained from all participants. All methods will be conducted in accordance with the ethical standards of the Declaration of Helsinki and in accordance with relevant guidelines and regulations. We will disseminate the findings through peer-reviewed publications and conference presentations and send them to the participants.

**Discussion:**

This trial will demonstrate which supplementary intervention can better improve the impact of a patient-led goal-setting approach to treat LBP. If successful, the results will potentially have implications for athletic trainers, physiotherapists, and health care practitioners.

**Trial registration:**

IRCT Iranian Registry of Clinical Trials IRCT20210927052616N1. Registered on November 03, 2021.

**Supplementary Information:**

The online version contains supplementary material available at 10.1186/s13063-023-07617-1.

## Strengths and limitations of this study

- This is the first study to investigate primarily the effect of the patient-led goal-setting approach and PNE compared with the patient-led goal-setting approach and MT on pain intensity, disability, and quality of life in patients with chronic LBP.
- The secondary aim of this study is to investigate the effect of patient-led goal-setting approach and PNE compared with the patient-led goal-setting approach and MT on psychological and psychological factors in patients with chronic LBP.
- This trial will demonstrate which supplementary intervention can better improve the impact of a patient-led goal-setting approach to treat LBP.
- If successful, the results will potentially have implications for athletic trainers, physiotherapists, and health care practitioners

## Introduction

Low back pain (LBP) is one of the most common causes of disability across the world [1]. More than 85% of patients with LBP receiving primary care have experienced pain chronification [2]. Chronic LBP contributes to a highly significant burden on society and individuals, e.g., health-related costs, individual quality of life, and disability across the world [3]. Due to physiological and psychological factors behind the chronic type of LBP, it is associated with limitations in daily routines, kinesiophobia, and disability.

Some of the most widely used therapies in chronic LBP include pharmacotherapy, invasive therapies, biophysical and electrotherapy, exercise therapy, and/or manual therapy [2]. Nowadays, the lack of treatment interventions covering different and complex factors of LBP is obviously observed. Therefore, getting better results of exercise therapy, factors like knowledge of pain neuroscience and pain education and the role of psychosocial/cognitive and behavioral characteristics in pain are essential.

Meanwhile, self-management program as an effective intervention includes a goal-setting approach motivating the individuals to change their behavior in order to achieve a better result [4]. In self-management programs, there are two important factors needed to be considered. Self-determination and self-efficacy using a self-management approach can be achieved and can help patients identify problem areas of personal relevance associated with their condition, establish achieving goals, and applicable strategies [4]. In a self-management approach, the patient is encouraged to independently take the steps towards goal achievement and increase their sense of confidence and ownership in the goal setting skills [4, 5]. However, as a multifactorial type of chronic LBP, previously, it has been widely shown that better results can be obtained when a specific intervention is combined with a supplementary one [3].

Pain neuroscience education (PNE) is a psychological-based supplementary intervention aiming to change patients’ understanding about their pain and unhelpful beliefs. In PNE, the patient is taught about the physiology of pain, nociceptive pain, displaying different parts of the body in the brain, pain-related changes in body perception, and the psychological dimensions of pain [6].

Furthermore, manual therapy (MT) is also one of the most cost-effective and low-risk treatments for chronic LBP [7]. MT can be defined as mobilization and manipulation techniques for relieving pain and musculoskeletal dysfunction. MT is also associated with neurophysiological effects such as changes in the activity of alpha motor neurons and autoimmune response systems and an increase in blood levels of endorphin and serotonin, which have been shown to occur throughout the nervous system through peripheral, spinal, and supra-spinal mechanisms [7, 8].

In the previous studies [9–11], most therapeutic approaches to the treatment of chronic LBP were consistent with biomedical models. In these models, the focus is on structural and biomechanical disorders. Recent studies have shown that pain has sensory, behavioral, and psychological components, and the association of chronic LBP with psychological, behavioral, and social factors has been established. As a result, new biopsychosocial models for the treatment of chronic pain have been introduced, but the implementation of these approaches is not yet common, and a very limited number of studies have examined the multidimensional treatment of chronic LBP. Therefore, because of the lack of evidence supporting the benefits of a combined cognitive-based intervention’s effect, this randomized control trial (RCT) will investigate the effects of a patient-led goal-setting approach combined with PNE or manual therapy in patients with chronic LBP. It is hypothesized that intervention combined with PNE can show a better result especially on psychological outcomes.

## Aims

Primary aim: Investigating the effect of a patient-led goal-setting approach and PNE compared with a patient-led goal-setting approach and MT on pain intensity, disability, and quality of life in patients with chronic LBP.

Secondary aim: Investigating the effect of a patient-led goal-setting approach and PNE compared with a patient-led goal-setting approach and MT on psychological factors in patients with chronic LBP.

## Methods

### Study design and setting

**Fig. 1:**
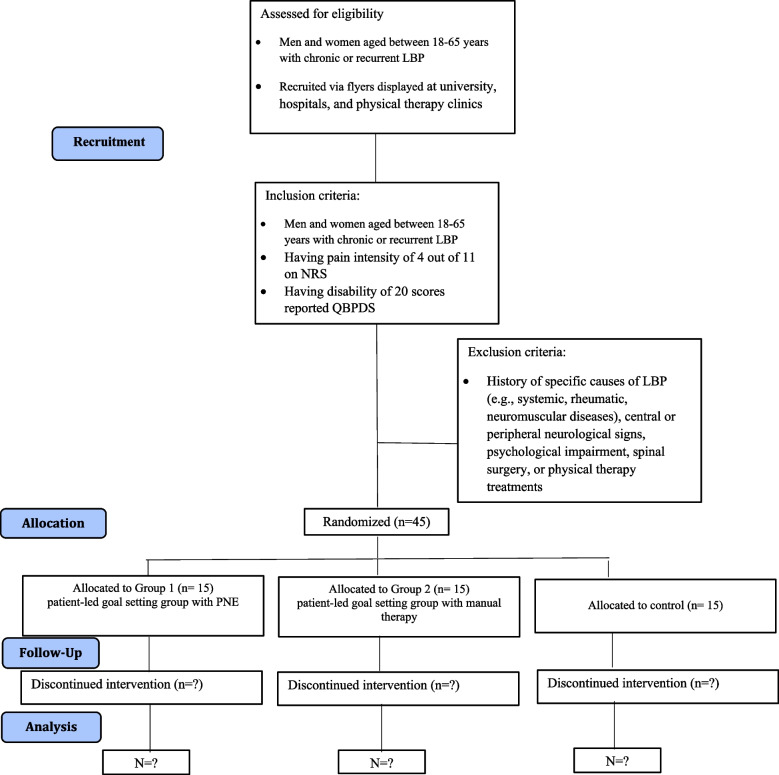
CONSORT diagram

This study is a three-arm single-blind RCT approved by the Ethics Committee in Research at Sport Sciences Research Institute of Iran (IR.SSRC.REC.1400.084) with the IRCT registration number IRCT20210927052616N1 and will be carried out in the Sport Medicine Laboratory of Kharazmi University of Iran. The CONSORT diagram for this study is provided in Fig. 1.

### Recruitment

We will recruit patients through practitioners via flyers distributed at Kharazmi University, hospitals, and physical therapy clinics. We will further use advertising posters in each center and online advertisements in the media. Then, participants will be in charge of screening for inclusion and exclusion criteria, and a final decision will be taken regarding the eligibility of the patients.

### Informed consent

Prior to the enrollment, all the participants will be fully informed about the objectives of the study and will provide written informed consent. Research assistants will obtain informed consent. The chief investigator retains overall responsibility for the informed consent of participants and will ensure that all those with delegated responsibility are authorized, trained, and competent to participate according to the protocol, principles of Good Clinical Practice (GCP), and Declaration of Helsinki.

### Eligibility criteria

The patients who participated in this study will be 105, both men and women aged between 18 and 65 years with chronic or recurrent LBP for > 3 months, pain intensity of 4 out of 11 Numerical Rating Scale (NRS), and at least 20 scores reported on the Quebec Back Pain Disability Scale (QBPDS) [5]. Those patients with a history of specific causes of LBP (e.g., systemic, rheumatic, neuromuscular diseases), central or peripheral neurological signs, psychological impairment, spinal surgery, or physical therapy treatments in the last 6 months prior to the baseline assessment will be excluded from the study. Criteria assessment will be performed by an experienced physical therapist.

### Allocation

Block randomization will be used in this study. A person not involved in the execution of the trial generates the randomization list using block randomization via computer-generated random numbers. The same person prepares sealed envelopes containing the treatment allocation information. The block size is not revealed to the study group before analyses. The envelopes are stored in a secure place at the study center. After receiving the informed consent, a researcher who did not apply the intervention (treatment approaches) or evaluate the outcomes gives a sealed envelope to the patient containing the treatment allocation information, and the treatment is arranged accordingly.

### Blinding

The blind researcher will be blinded from the treatment allocation when collecting the outcome measurements. The patient will be wearing long-covered clothes during the follow-up visits and asked not to reveal the given treatment. The blinding of the treatment approach is not possible for the personnel executing the treatment approach nor the patients. This study will be conducted as a pre-test/post-test design with a control group and blinded assessor and data analyst.

### Description of the interventions

#### Patient-led goal-setting approach

The intervention will be carried out for 8 weeks including 5 face-to-face sessions. The first session takes about 1 h, and the other sessions are held for 15 to 30 min with 2-week intervals. Then, two follow-up sessions (lasting 30 min each) will be developed with a 1-month interval; furthermore, one session will be organized 12 months after the first session, and finally, the results will be collected. The SMART model will be used for applying this intervention. This model includes a special process for goal setting that is measurable, achievable, and associated to a specific time return. In this model, the researcher is trained in setting goals by the patient, considers the patient’s history of LBP, and discusses about the issue that led to the occurrence of LBP [4]. Patients are asked to prioritize these problems based on what they want to focus on. Then, the strategies are discussed based on evidence-based guidelines, and the patient sets specific goals and strategies to work independently between sessions. Patients record their set goals, progress towards these goals, and agree on strategies for achieving the goal in their workbooks. If the goal strategies include consultation with a health care professional, the patient is encouraged to pursue this independently (Table 1) [3–5].

**Table 1:** Components of the interventions

Patient-led goal-setting intervention
Session 1: week 1
► Orientation of patient
► SMART approach explained
► Goals and strategies developed
Homework task: review educational material
Session 2: week 3
► Education and discussion
► Review of goals, progress towards, and barriers to achieving goals
► Strategies developed
Sessions 3 and 4: weeks 5 and 7
► Review of goals, progress towards, and barriers to achieving goals
► Strategies developed
Session 5 (completion of the intervention): 2 months
► Review of goals, progress towards, and barriers to achieving goals
► Strategies developed
► Outcomes measured
Post-intervention follow-up (3 months)
► Review of goals, progress towards, and barriers to achieving goals
Post-intervention follow-up (4 months)
► Review of goals, progress towards, and barriers to achieving goals
► Outcomes measured
Post-intervention follow-up (12 months)
► Outcomes measured

#### Pain neuroscience education

It includes 4 sessions being organized once a week for 40 to 45 min in person and in each session issues such as neuropathic pain, allodynia, central sensitization, hyperalgesia, peripheral nerve sensitization, neuroplasticity, spreading pain, stress biology, immune response, hypersensitivity, fear, tragedy and pain, and how to cope with pain will be discussed to patient through pictures, terms, and descriptions (Table 2) [12].

**Table 2:** The content of neuroscience pain education

1 session	Peripheral neuropathic pain, peripheral nerve sensitization, allodynia, central sensitization, hyperalgesia
2 sessions	Neuroplasticity, spreading pain, central sensitization, hyperalgesia, allodynia
3 sessions	Stress biology, immune response, emotional overload, fear, catastrophization, pain
4 sessions	How to cope with pain?

#### Manual therapy

MT includes 18 sessions of treatment for 6 weeks lasting 60 min each. Soft tissue mobilization, muscle energy techniques, and joint mobilization will be planned to be performed. Soft tissue mobilization includes myofascial stretching for superficial and deep muscles and transverse friction for intervertebral and supraspinal ligaments. Muscle energy techniques include relaxation after isometric contraction for the quadratus lumborum and piriformis muscles that we ask patients to gently contract for 8 s at 30% of maximum voluntary contraction force, and this movement will be repeated after each rest interval. Joint mobilization will be evaluated based on sacroiliac mobility test with standing flexion forward test, Gillet test, and Piedallu Sign test [7, 8].

### Description of the control

Participants in the control group will receive no specific intervention. However, for meeting the ethical codes, we will provide the patients in the control group with a pamphlet included with useful information on taking care during chronic LBP.

### Outcome measures

All the outcome measures explained below were recorded at baseline (pre-test), 2 months post-test, and 4 and 12 months follow-up time points. The flow of the study is also provided in Table 3.

**Table 3:** Schedule of enrollment, intervention, study visits, and assessments for all three study groups

Time point	Study period													
	Enrollment	Allocation	Patient-led goal-setting group with PNE				Patient-led goal-setting group with MT				Control group			
			Start	2 m	4 m	12 m	Start	2 m	4 m	12 m	Start	2 m	4 m	12 m
Enrollment														
Eligibility screen	*													
Informed consent	*													
Baseline assessment	*													
Allocation		*												
Intervention														
Patient-led goal-setting group with manual therapy							*	*	*	*				
Patient-led goal-setting group with PNE			*	*	*	*								
Assessments														
Start back			*				*				*			
QBPDS			*	*	*	*	*	*	*	*	*	*	*	*
NRS			*	*	*	*	*	*	*	*	*	*	*	*
SF_36			*	*	*	*	*	*	*	*	*	*	*	*
DASS			*	*	*	*	*	*	*	*	*	*	*	*
PSEQ			*	*	*	*	*	*	*	*	*	*	*	*
FABQ			*	*	*	*	*	*	*	*	*	*	*	*
Tampa Scale			*	*	*	*	*	*	*	*	*	*	*	*
PCS			*	*	*	*	*	*	*	*	*	*	*	*
RNPQ			*	*	*	*	*	*	*	*	*	*	*	*
CSI			*	*	*	*	*	*	*	*	*	*	*	*
Adverse events			*	*	*	*	*	*	*	*	*	*	*	*

*PNE* pain neuroscience education, *MT* manual therapy, *NRS* Numerical Rating Scale, *QBPDS* Quebec Back Pain Disability Scale, *PSEQ* Pain Self-Efficacy Questionnaire, *DASS* Depression Anxiety Stress Scale, *FABQ* Fear Avoidance Beliefs Questionnaire, *TSK* Tampa Scale for Kinesiophobia, *PCS* Pain Catastrophizing Scale, *CSI* Central Sensitization Inventory

#### Demographic data

The sociodemographic data will include gender, age, weight, height, BMI, back pain intensity recorded by the Start Back Questionnaire, LBP history, level of education, and employment situation.

#### Start Back Questionnaire

The Start Back Questionnaire is going to classify patients’ state of pain. The Start Back (9-item version) is a brief validated tool, designed to screen primary care patients with LBP for prognostic indicators that are relevant to initial decision-making [13].

### Primary outcomes

#### Pain intensity

A Numeric Rating Scale (NRS) will be used as a reliable tool (ICC = 0.83) in order to measure pain intensity in eligible patients [14]. NRS will be numbered from 0 to 10, in which patients will rate their pain from 0 (painless) to 10 (worst imaginable pain) [14].

#### Disability

Disability will be assessed using the Quebec Back Pain Disability Scale (QBPDS) (minimum score = 20 and maximum score = 100) [3]. This questionnaire is about how LBP affects people’s daily living, which includes 20 items. The reliability of this questionnaire has been reported by researchers in previous research at different time intervals (ICC = 0.80) [15].

### Secondary outcomes

#### Quality of life

Quality of life is evaluated using the SF-36 questionnaires. SF-36 is a reliable (ICC = 0.80), multipurpose, short-term health survey with 36 questions and is the most widely used public health standard. The scores of each scale vary from 0 to 100, 0 reporting the worst and 100 reporting the best conditions on the scale [16].

#### Depression, anxiety, and stress

To evaluate these psychological outcomes, the Depression Anxiety Stress Scale (DASS) will be used. DASS consists of 21 questions in which each psychological factor is evaluated by 7 questions: depression (range 0–28), anxiety (range 0–20), and stress (range 0–34). A higher score indicates greater intensity. DASS reliability has been observed previously as ICC = 0.88 [17].

#### Self-efficacy

Self-efficacy will be assessed using the Pain Self-Efficacy Questionnaire (PSEQ). This questionnaire is a reliable tool (ICC = 0.82) which includes 10 items. Each item questions the patients’ assessment of their own ability to perform a battery of activities despite pain on a 7-point Likert scale (0 to 6). Higher scores will indicate greater self-efficacy [18].

#### Fear-avoidance beliefs

Fear-Avoidance Beliefs Questionnaire (FABQ) is a 16-item scale describing how much fear and avoidance affect patients, and it also determines what kind of psychosocial interventions are effective for these patients (ICC = 0.90) [19].

#### Kinesiophobia

Kinesiophobia is measured by the reliable Tampa Scale for Kinesiophobia (TSK) (ICC = 0.70). TSK is a 17-item tool, and each item is answered as a 4-point Likert scale. The final score on the scale is between 17 and 68 [20].

#### The Pain Catastrophizing Scale

The Pain Catastrophizing Scale (PCS) is a self-report reliable questionnaire that assesses inappropriate coping strategies and catastrophic thinking about pain and injury (ICC = 0.90). This 13-item scale will be used to assess the range of catastrophic thoughts and behaviors of patients when faced with pain and consists of three subscales of mental rumination, exaggeration, and despair. In previous studies, it shows an average score of 18 in healthy individuals, while in patients with pain, this number is higher and a score of more than 30 has been reported [21].

#### Pain neurophysiology

The Revised Pain Neurophysiology Questionnaire (RNPQ) with 0.84 of ICC will be used for measuring pain neurophysiology. It has 13 items. The scores range from 0 to 13. Higher scores indicate a higher level of neuroscience knowledge [22].

#### Central sensitivity

Central sensitivity will be assessed using the Central Sensitization Inventory (CSI) scale (ICC = 0.91). The CSI includes 25 questions identifying and quantifying the main symptoms associated with Central Sensitivity Syndrome. A score of more than 40 points is reported as a clinical indicator [23].

### Plans to promote participant retention and complete follow-up

To ensure an adequate follow-up rate, the research team will:

- Maintain regular contact with participants after giving informed consent (regularly every 4 weeks)
- Ensure contact occurs in the 4 weeks prior to the baseline assessment (pre-test), to ensure greater levels of contact between consent and the intervention starting
- After randomization, participants will be given the opportunity to meet a specific therapist and ask questions prior to the intervention starting
- Participants will be reimbursed for their transportation expenses

### Improve adherence to the intervention protocols where an intervention appointment is missed

- The research assistant will follow up non-attendees via telephone call to ascertain the reason for non-attendance and whether any assistance from the study team can help in this matter.
- The research assistant will check if the participant is still willing to continue with the appointed intervention and the study time points outcome measure assessments. In case a participant discontinued the intervention protocol for more than 3 sessions, they would not be included in the analysis.

### Sample size estimation

Sample size has been estimated via G*Power 3.1.9.4 based on the therapy effects on pain and accounting for a 10% loss to follow-up after 4 and 12 months. An a priori power analysis was performed considering full factorial repeated measures and using a small effect size (Cohen’s *d*) of 0.25, confidence level (*α* = 0.05), and desired power (95%). The required sample size was calculated as 15 participants per group. Table 3 provides a schedule of enrollment, intervention, study visits, and all the assessments performed for all three study groups.

### Statistical analysis

The data will be summarized with descriptive statistics (mean, median, standard deviation, percentiles for numerical variables, frequencies and percentages for categorical variables). The SPSS software version 26 will be used to analyze the data. The Shapiro–Wilk test will be used to examine the normal distribution of data. If the normal distribution is approved, the mix model ANOVA will be used to compare the differences in scores related to each variable in the pre-test and post-test in each group (with-in group effects) and to examine the differences between groups in each time point (between group effects), and Bonferroni post hoc test will be used to find significant differences and compare the groups. This model allows for possible missed data. We will assume data missing at random. Study group and time of assessment will be used as fixed factors and patients will be used as random factors. Otherwise, the Mann–Whitney *U* test will be used to compare two independent groups in case the normal distribution was not approved. Finally, after post hoc tests, the Cohen test (*d*) will be used in order to calculate the effect size [24]. *P*-value < 0.05 will be considered significant. The main conclusion will be drawn from the unadjusted analysis without performing any subgroup analysis of primary and secondary outcomes.

### Data monitoring

Data monitoring committee, interim analyses, or stopping guidelines are not included in this study because all the treatment approaches applied in this study are already in daily practice and the results have been acceptable. However, any unexpected adverse events that occurred during the intervention period will be reported to a highly experienced physiotherapist who will not be involved in the execution of the trial. This physiotherapist will be available to make the final decision to terminate the trials in case of unanticipated harm.

### Harms

No potential harm is anticipated for the participants of this study. All complications and harms (in case of any) will be reported to a highly experienced physiotherapist who will be available to make the final decision to terminate the trial participation for a participant in case of unanticipated harm. All the complications and harms (in case of any) will be reported also to the Research Ethics Committee. Major and minor complications (in case of any) will be listed in the safety consideration section.

### Protocol amendments

In the case of modification of the study protocol, all changes will be updated to the Iranian Registry of Clinical Trials.

### Confidentiality

Trial data will be stored in a secure storage at the study center for 10 years after the completion of the study. All data will be handled based on the reasonable request from the corresponding author.

### Implementation

The recruitment is done by a researcher who is not involved in the execution of the treatment approaches and the outcome measure analysis. After receiving the written consent, this researcher opens the envelope, and the patient is then randomized to one of the study groups. A blinded assessor (S.S.) carries out the baseline measures, and the patient receives a written guide for the treatment approach.

### Patient and Public Involvement

Although participants of two groups of this study will actively be involved in the treatment procedures of patient-led goal-setting approach and pain neuroscience education groups, there will be no direct patient or public involvement in the study design. Patients, therefore, will not be invited to comment on the study design. The overall study design of this study was developed from previous experience of the investigators involved in the design and coordination of similar studies. Any possible burden of the treatment programs and standard care will be assessed fortnightly throughout the trial. A written summary of the results will be disseminated to participants at the end of the study. Following their enrollment in the trial, participants can request to receive a copy of their assessments after finishing the study if required for allied health or medical interventions. Finally, patients will not be invited to contribute to the writing or editing of any possible manuscript out of the findings of this study for readability or accuracy.

### Ancillary and post-trial care

Patients will be treated during and after the trial with best intentions. If malpractice has taken place, patients will be compensated by the chief researcher.

## Discussion

The aim of this study will be to compare the effect of patient-led goal-setting approach combined with PNE or MT in patients with chronic LBP. The main question of this study is whether the patient-led goal-setting approach combined with PNE is more effective than MT in reducing pain intensity related with chronic LBP. Further objectives will be also to assess the effect of these two interventions on disability, quality of life, and psychosocial factors in chronic LBP compared to a control group. A total of 105 patients will participate in this study and the athletic trainer who performs the pre-test and post-test as well as follow-up evaluations will be blinded to the participants’ group code. It is expected that this RCT will provide novel data on the effectiveness of the patient-led goal-setting approach combined with PNE or MT in patients with chronic LBP compared to a control group.

In the current study, we will evaluate the effects of combined interventions on psychological outcomes as well as central sensitivity. It is expected that this prospective trial may contribute towards refining guidelines for good clinical practice and may be used as a basis for health authorities’ recommendations. If successful, the findings and information provided by this study will potentially have implications for physiotherapists, athletic trainers, and health care practitioners.

## Trial status

This is version 2.0 of this clinical trial protocol, dated June 04, 2022. Recruitment for the trial began on October 22, 2021, under protocol version 1.0, dated November 24, 2020. Recruitment is expected to be completed in the first quarter of 2023. Trial Status is “in progress.”

### Supplementary Information

**Additional file 1.**

## Data Availability

Data will be available from the corresponding author on a request once it has been collected.

## Abbreviations

LBP: Low back pain
RCT: Randomized control trial
PNE: Pain neuroscience education
MT: Manual therapy
GCP: Good Clinical Practice
NRS: Numerical Rating Scale
QBPDS: Quebec Back Pain Disability Scale
PSEQ: Pain Self-Efficacy Questionnaire
DASS: Depression Anxiety Stress Scale
ICC: Intra-class correlation coefficient
FABQ: Fear Avoidance Beliefs Questionnaire
TSK: Tampa Scale for Kinesiophobia
PCS: Pain Catastrophizing Scale
RNPQ: Revised Pain Neurophysiology Questionnaire
CSI: Central Sensitization Inventory
